# Dimensionality tuning of the electronic structure in Fe_3_Ga_4_ magnetic materials

**DOI:** 10.1038/srep28364

**Published:** 2016-06-22

**Authors:** K. O. Moura, L. A. S. de Oliveira, P. F. S. Rosa, C. B. R. Jesus, M. E. Saleta, E. Granado, F. Béron, P. G. Pagliuso, K. R. Pirota

**Affiliations:** 1 Instituto de Física “Gleb Wataghin”, Universidade Estadual de Campinas (UNICAMP), Campinas-SP, 13083-859, Brazil; 2 Núcleo Multidisciplinar de Pesquisa, Universidade Federal do Rio de Janeiro (UFRJ) - Campus Xerém, Duque de Caixias-RJ, 25245-390, Brazil; 3 Brazilian Synchrotron Light Laboratory (LNLS)/Brazilian Center of Energy and Materials (CNPEM), Campinas-SP, 13083-970, Brazil

## Abstract

This work reports on the dimensionality effects on the magnetic behavior of Fe_3_Ga_4_ compounds by means of magnetic susceptibility, electrical resistivity, and specific heat measurements. Our results show that reducing the Fe_3_Ga_4_ dimensionality, via nanowire shape, intriguingly modifies its electronic structure. In particular, the bulk system exhibits two transitions, a ferromagnetic (FM) transition temperature at *T*_1_ = 50 K and an antiferromagnetic (AFM) one at *T*_2_ = 390 K. On the other hand, nanowires shift these transition temperatures, towards higher and lower temperature for *T*_1_ and *T*_2_, respectively. Moreover, the dimensionality reduction seems to also modify the microscopic nature of the *T*_1_ transition. Instead of a FM to AFM transition, as observed in the 3D system, a transition from FM to ferrimagnetic (FERRI) or to coexistence of FM and AFM phases is found for the nanowires. Our results allowed us to propose the magnetic field-temperature phase diagram for Fe_3_Ga_4_ in both bulk and nanostructured forms. The interesting microscopic tuning of the magnetic interactions induced by dimensionality in Fe_3_Ga_4_ opens a new route to optimize the use of such materials in nanostructured devices.

Nanowires belong to a new class of quasi-unidimensional materials that have been attracting great interest in the last few years due to their numerous multidisciplinary potential applications, such as functional materials in biomedical sciences^1^, electronics^2^, optics^3^, magnetic devices^4^ and energy storage^5^. Among the several procedures developed for nanowire systems synthesis, it is noteworthy to mention template-assisted fabrication methods^6^, vapor-liquid-solid mechanism^7^, molecular beam epitaxy^8^ and electrochemical nanolithography^9^. In particular, nanoporous alumina membranes have been widely used as templates for magnetic nanowire arrays produced by electrochemical deposition due the simplicity, versatility, efficiency and low cost implementation of this technique. However, the nanowires obtained by this method generally present poor crystallinity and are restricted to metallic alloys.

Recently, the novel metallic-flux nanonucleation (MFNN) technique has been successfully developed to nucleate crystalline nanowires inside alumina membrane pores^10^,^11^. The nanoporous template presents several advantages, such as an excellent pore size control over large areas (obtained by varying the oxidation conditions), as well as pores with large aspect ratio that exhibit a spatial distribution with a highly regular pattern. Therefore, by using an alumina template during a metallic flux growth, the MFNN technique allows to confine the crystalline compounds into a quasi-1D shape. In addition to the high probability to obtain single crystal nanowires, this technique opens opportunities to fabricate novel intermetallic compounds in nanowire shape, besides the advantage of simultaneously obtaining both systems (bulk and nanowires)^10^,^11^. In this regard, it is extremely desirable to develop alternative techniques to synthesize a wide range of high quality crystalline nanowires.

In this work we present the magnetic characterization of the Fe_3_Ga_4_ intermetallic compound synthesized by the MFNN technique in both bulk and nanowire forms. As determined by Philippe *et al*. Fe_3_Ga_4_ presents a complex base-centered monoclinic structure with eighteen Fe atoms per unit cell occupying four non-equivalent sites^12^. It has also been observed that the bulk compound exhibits a complicated magnetic behavior. At low magnetic fields, a ferromagnetic (FM) state develops below temperature *T*_1_ = 50 K, while at higher temperatures, antiferromagnetism (AFM) takes place before vanishing at a Néel temperature *T*_2_ = 390 K^13^. This behavior has been explained by Moriya and Usami’s theory, which predicts coexistence of FM and AFM states in itinerant electron systems^13^,^14^. To investigate these peculiar magnetic and structural behaviors, several chemical substitution studies have been performed on both Fe and Ga sublattices^15^,^16^,^17^,^18^. In particular, it has been recently reported that when grown by the alternative deposition of Fe_3_GaAs and GaAs on a GaAs (001) substrate, the Fe_3_Ga_4_ compound presents a distinctive photo-enhanced magnetization at room temperature^19^. On the other hand, Fe-Ga nanowires, with different composition from that studied in this work, have been successfully fabricated by electrochemical deposition and have been widely investigated for potential use as sensing elements in a variety of microelectromechanical and nanoelectromechanical-based biomimetic devices^20^,^21^. Therefore, it is very instructive to study the dimensionality effects on Fe_3_Ga_4_ interesting properties. To the best of our knowledge, no such studies about Fe_3_Ga_4_ compound have not yet been reported in the literature.

In this work, we report the dimensionality effects on the Fe_3_Ga_4_ magnetic field-temperature phase diagram (*H*–*T*), which was constructed for both bulk and nanowire systems using magnetization, specific heat and electrical resistivity measurements. The results are discussed within the framework of the Moriya and Usami’s theory on magnetic phase transitions in itinerant electron systems. As such, this work reveals unambiguous evidence for a dimensionality tuning of the Fe_3_Ga_4_ electronic structure that modifies the microscopic exchange parameters considered in Moriya and Usami’s theory. More generally speaking, we strongly believe that the possibility of growing intermetallic compounds in nanowire form will open an interesting branch in the understanding of fundamental properties, as well as permitting to control the nanowire characteristics for desired applications.

## Results and Discussion

### Morphology and Composition

**Figure 1:**
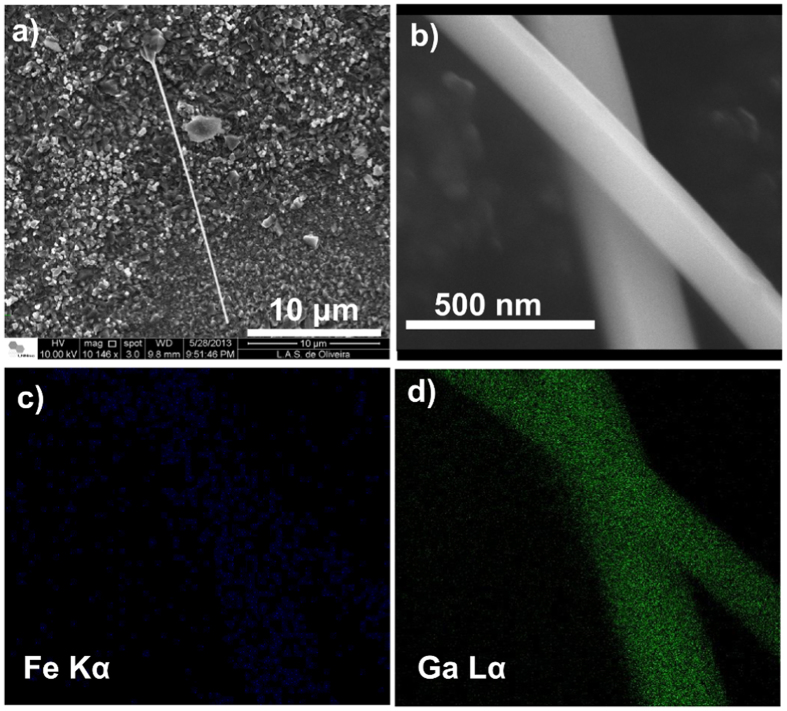
SEM images of **(a)** an isolated nanowire, **(b)** two nanowires of Fe_3_Ga_4_. EDS composition mapping related to **(c)** Fe K*α* and **(d)** Ga L*α* energies.

Scanning electron microscopy (SEM) was first performed to verify the presence, dimensions and composition of the Fe_3_Ga_4_ nanowires. As shown in Fig. 1(a), most of the observed isolated nanowires exhibit a diameter of ~250 nm and a length of ~25 *μ*m, giving a length/diameter aspect ratio of around 100. Figure 1(b) displays a magnified view of two nanowires showing their surface quality by presenting a very low roughness and no apparent defects. Its energy dispersive X-ray spectrometry (EDS) mapping for Fe *Kα* and Ga *Lα* energies, shown respectively in Fig. 1(c–d), clearly states that both Fe and Ga elements are present in the nanowires. However, the quantitative chemical composition given by EDS was not used since the analyzed surface was not polished and that Fe has a weak detection power compared to Ga, due to its low atomic number.

### Structural Characterization

X-ray diffraction (XRD) data were used to identify the obtained bulk crystalline phase. The measurements were performed using Cu *Kα* radiation and *θ*–2*θ* scans were recorded in the 25°–55° range. From the study of the X-ray diffraction pattern we can affirm that the Fe_3_Ga_4_ phase was formed in the expected *C*2/*m* (#12) space group. The obtained crystals present a higher crystalline quality compared to those fabricated via arc melting^13^. Furthermore, no formation of additional phases was observed, such as FeGa_3_, which is commonly observed during Fe_3_Ga_4_ growth^22^.

On the other hand, the nanowires structural characterization required synchrotron X-ray diffraction and absorption measurements, both performed at the Brazilian Synchrotron Light Laboratory (LNLS). The main difficulty encountered arises from the alumina membrane present around the nanowires, the latter only forming a small percentage of the measured system.

**Figure 2:**
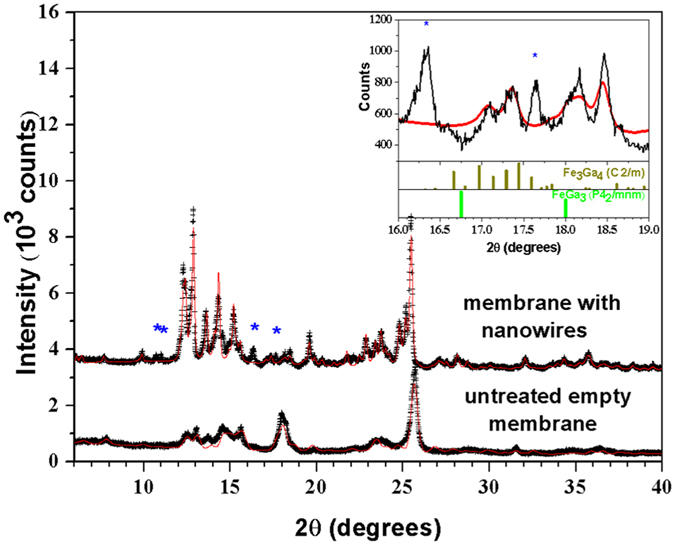
Experimental X-ray diffraction profiles for the untreated empty membrane and for the embedded nanowires sample (translated along the vertical axis for clarity), taken with *λ* = 0.6199 Å. The red line for the untreated membrane is a Rietveld fit using a model with an admixture of *η*- and *θ*-Al_2_O_3_ phases (space groups *Fd*-3*m* and *C*2/*m*, respectively). The red line for the embedded nanowires sample is a Le Bail fit using two monoclinic phases with *C*2/*m* space group. Inset: Selected portion of the X-ray diffraction profile for the embedded nanowires sample. The red line shows the results of the Le Bail fit for the Al_2−*x*_Ga_*x*_O_3_ phases. The positions and intensities for the expected Bragg peaks of the Fe_3_Ga_4_ and FeGa_3_ binary phases in this regions are indicated as vertical bars. Unidentified peaks are marked with blue asterisks.

In a first step, we used the X-ray Diffraction and Spectroscopy (XDS) beamline to acquire the XRD spectra of the membrane with embedded nanowires, as well as the untreated empty porous alumina membrane. The data were collected in transmission mode at room temperature and atmospheric pressure with a wavelength of 0.6199 Å. The spot size was 2.0 × 0.2 mm^2^. As we can see in Fig. 2, the diffraction profile of the empty membrane could be reasonably well modeled with the program suite GSAS+EXPGUI^23^,^24^ by an admixture of *η*- and *θ*-Al_2_O_3_ phases (space groups *Fd*-3*m* and *C*2/*m*, respectively)^25^, with no clear sign of contamination with impurity phases. However, in the treated membrane with nanowires, the cubic *η*-Al_2_O_3_ phase was not clearly observed. Instead, the main features of the observed profile could be modeled by a mixture of two *θ*-Al_2_O_3_ phases with distinct lattice parameters and ~4/1 proportion in the corresponding Bragg peak intensities. The observed lattice parameters, obtained from a Le Bail fit of the observed profile, are a = 11.918(1) Å, b = 2.9535(3) Å, c = 5.6864(4) Å, and *γ *= 103.92(1)° for the majority phase, and a = 12.052(6) Å, b = 2.998(1) Å, c = 5.771(3) Å, and *γ *= 103.44(5)° for the minority one. While the refined unit cell volume of the *θ*-Al_2_O_3_ phase of our untreated membrane (V = 187.3(1) Å^3^) agrees well with the literature value for *θ*-Al_2_O_3_ (V = 187.4–187.9 Å^3^ 25,26), it is not the case for the treated membrane phases (V = 194.28(2) Å^3^ and V = 202.8(1) Å^3^, respectively). However, their comparison with unit cell volume values from the literature for isostructural *β*-Ga_2_O_3_ (V = 209.0–209.5 Å^3,^^27^,^28^) indicates that the Al ions in the membrane are partly replaced by Ga during the Fe_3_Ga_4_ crystal growth procedure. This leads to an inhomogeneous solid solution with an Al_1.4_Ga_0.6_O_3_ majority phase and an Al_0.6_Ga_1.4_O_3_ minority phase, where the respective compositions of the membrane phases were estimated from the unit cell volumes through the Vegard’s law. Extra Bragg peaks due to additional polycrystalline phases were also observed in the membrane with embedded nanowires profile, with intensities of ~10% or lower with respect to the strongest peak of the main monoclinic Al_1.4_Ga_0.6_O_3_ membrane phase. These extra peaks could not be related to any known phase of the Fe-Ga binary system, and most likely arise from additional phases of the Al_2−*x*_Ga_*x*_O_3_ membrane. Efforts towards an unambiguous identification of the minor membrane phases were unsuccessful, which is justified considering the possibilities of compositional fluctuations that would shift the Bragg peaks with respect to the expected angles, preferred orientation of membrane phases and the large variety of possible Al_2−*x*_Ga_*x*_O_3_ phases. In any case, neither Fe_3_Ga_4_ nor any other known Fe-Ga binary compound such as FeGa_3_^29^ were unambiguously identified in the membrane with nanowires diffractogram (see Fig. 2 inset), meaning that the weight fraction of any such metallic phases within the membrane must be below 2%. This conclusion is consistent with our SEM analysis, which also indicated a low filling factor of the pores. We should mention that, while the Al ions in the membrane are partly replaced by Ga during the Fe_3_Ga_4_ crystal growth procedure, we cannot discard the possibility that, conversely, some Ga ions in the crystals are partly replaced by Al^18^.

**Figure 3:**
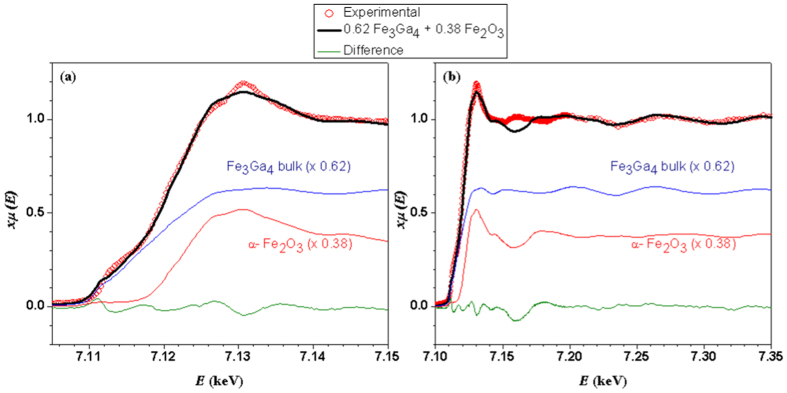
Fe K-edge X-ray absorption spectrum of membrane with embedded nanowires, bulk Fe_3_Ga_4_ (x 0.62) and *α*-Fe_2_O_3_ (x 0.38); **(a)** near-edge region and **(b)** extended spectra. The thick black lines represent the weighted sum of the Fe_3_Ga_4_ and *α*-Fe_2_O_3_ spectra and the thin green lines show the difference between this combined spectra of the standard samples and the experimental data for the membrane with embedded nanowires system. The XANES fitting range was 7092–7147 eV.

In order to obtain structural information of the phases containing Fe, an element-specific technique became necessary. X-ray absorption measurements were therefore performed at the Fe K-edge in fluorescence mode at the XAFS2 beamline on the embedded nanowires, while bulk Fe_3_Ga_4_ and hematite (*α*-Fe_2_O_3_) were taken as standard samples. The near-edge region of the spectrum (XANES) is shown in Fig. 3(a), whereas a more extended region up to 7350 eV, covering the first extended X-ray absorption fine structure (EXAFS) oscillations, is displayed in Fig. 3(b). The prominent XANES peak at 7130 eV found for the embedded nanowires system indicates the presence of oxidized Fe^3+^ ions, while the strong pre-edge feature at 7112 eV is consistent with the presence of a significant fraction of non-oxidized Fe. The observed spectrum could be fitted by a combination of the spectra from Fe_3_Ga_4_ (62(1)%) and *α*-Fe_2_O_3_ (38(1)%), with good agreement both in near-edge region (Fig. 3(a)) and for first EXAFS oscillations above 7170 eV (Fig. 3(b)). Based on this analysis, we attribute the presence of oxidized Fe^3+^ in our membrane with nanowires sample to a small degree of substitution of Fe atoms into the Al_2−*x*_Ga_*x*_O_3_ membrane, while the major metallic Fe component found in our XAS spectrum is attributed to the Fe_3_Ga_4_ nanowires. Despite the good overall agreement provided by our simple analysis using Fe_2_O_3_ and Fe_3_Ga_4_ standards, a clear discrepancy between the observed spectrum for the embedded nanowires sample and the mixture of standards can be seen at ~7160 eV. This may be attributed to a distinct non-local electronic structure of *α*-Fe_2_O_3_ with respect to Fe^3+^ ions impurity into the Al_2−*x*_Ga_*x*_O_3_ membrane, which may lead to large energy shifts of high-energy XANES excitations involving charge transfer from Fe to neighboring ions.

### Magnetic Characterization

In order to investigate the magnetic properties and build the magnetic phase diagram of the Fe_3_Ga_4_ bulk and nanowire array, magnetization measurements were acquired both as a function of the temperature *T* (2–400 K) and the applied magnetic field *H* (±20 kOe). For the nanowire array characterization, the magnetic field was applied parallel to the nanowires axis.

**Figure 4:**
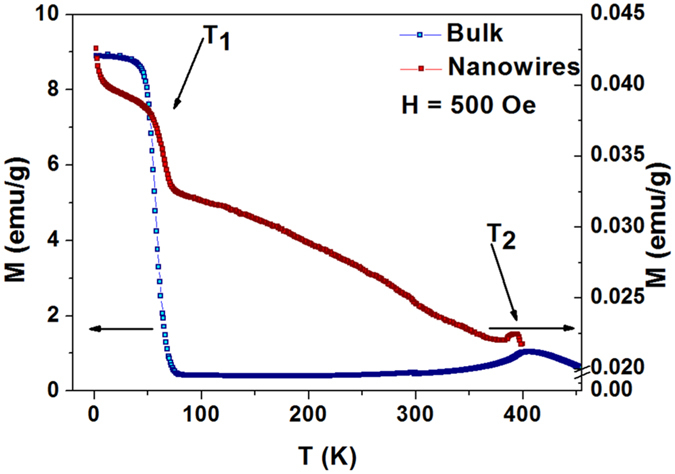
Temperature-dependent magnetization curves for Fe_3_Ga_4_ bulk (blue symbols, left axis) and nanowire array (red symbols, right axis) with an applied field of 500 Oe.

From the magnetization as a function of temperature curves, the two expected magnetic phase transitions are distinguished in terms of a magnetization drop around 50 K (*T*_1_) and a peak around 390 K (*T*_2_). In the case of nanowires, the reduced dimensionality affects *T*_1_ and *T*_2_ values in opposite ways. As we can see in Fig. 4, *T*_1_ increases while *T*_2_ decreases. Moreover, the relative magnetization drop at the first temperature transition *T*_1_ is smaller for the nanowire array compared to the bulk one. This could suggest that we are dealing with a phase transition from a FM to another magnetic phase order with net macroscopic magnetization in the nanowire case (FERRI or coexistence of FM and AFM). As discussed later, this phase coexistence is predictable from Moriya’s theory. This is the first evidence that the nature of this transition differs in the nanowires and in the bulk samples. We also observe that the temperature-dependent magnetization curves exhibit appreciable thermal hysteresis around *T*_1_, but only in the Fe_3_Ga_4_ nanowires case (not shown here), which decreases when we increase the applied magnetic field. These effects persist for all magnetic field values used in the magnetization measurements, and are characteristic of first order phase transition. In the case of bulk samples, no thermal hysteresis is noticed. Furthermore, we can see that the magnetic signal from Fe_3_Ga_4_ phase (in emu/g) is about 500 times smaller that for the bulk crystal, which is consistent with our estimative by XRD that the weight fraction of the Fe_3_Ga_4_ phase in the membrane is below 2%.

**Figure 5:**
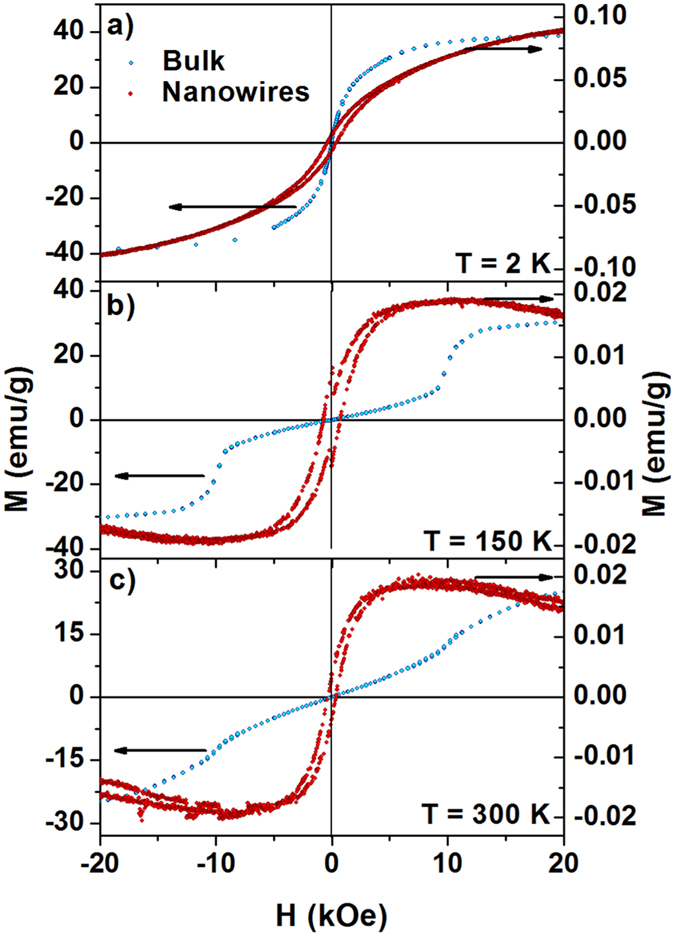
Field-dependent magnetization curves for Fe_3_Ga_4_ bulk (blue symbols, left axis) and nanowire array (red symbols, right axis): **(a)** 2 K **(b)** 150 K **(c)** 300 K.

Between 50 and 300 K, we observe a field-induced transition on the field-dependent magnetization curves for the bulk compound (Fig. 5). On the other hand, no such transition is detected for the nanowire system, but rather a large magnetic susceptibility and the presence of a magnetic hysteresis between 2 and 300 K, also supporting the FERRI or coexistence FM and AFM behavior suggested before. The magnetic results for bulk Fe_3_Ga_4_ are in good agreement with those obtained by Kawamiya and Adach^13^ and interpreted based on the Moriya and Usami’s theory for magnetic phase transition in itinerant electron systems. The theory takes into account the coexistence of uniform and staggered magnetization using a Landau free energy expression, which contains both magnetization terms (up to the fourth power) and the coupling terms between both magnetization components^14^. In the absence of magnetic anisotropy, the relative magnitudes of the free energy coefficients give rise to four different magnetic phase diagrams, one of which corresponding to the experimentally obtained behavior for bulk Fe_3_Ga_4_.

### Specific Heat Characterization

**Figure 6:**
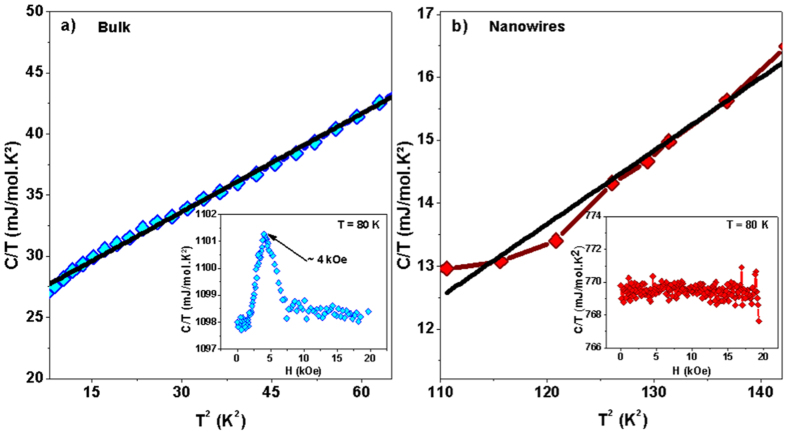
*C*/*T* vs *T*^2^ plot for Fe_3_Ga_4_ (a) bulk and (b) nanowires, where the black line represents a linear fit. The insets show field-dependent specific heat measurements for **(a)** bulk and **(b)** nanowires at 80 K.

Specific heat (*C*) measurements were performed in the 2–100 K and 2–20 kOe ranges of temperature and magnetic field, respectively. As shown in Fig. 6(a,b), the linear fits of the *C*/*T* versus *T*^ 2^ curves at low temperatures give similar Debye temperatures (*θ*_*B*_ = 230(2) K and *θ*_*N*_ = 253(15) K for bulk and nanowires, respectively). Since *θ* is related to the phonon spectrum, which in turn is related to the material crystalline structure, this result demonstrates that the nanowire array exhibits the same crystalline structure than that of Fe_3_Ga_4_ bulk phase. Moreover, we observe a reduction in the contribution of the conduction electrons to the specific heat when the system dimensionality is reduced. Remarkably, the electronic coefficient (*γ*) drops from 25.60 mJ/mol.K^2^ to almost zero (within experimental error), indicating that the Fe_3_Ga_4_ compound tends to become insulating in the nanowire morphology. These data are consistent with the magnetization results, since that a FERRI or FM behavior is most likely to be expected in localized electron magnetism. In addition, field-dependent specific heat measurement performed at 80 K shows a maximum at about 4 kOe for bulk Fe_3_Ga_4_ (see inset Fig. 6(a)), which corresponds to the field transition exhibited in magnetization hysteresis curves. Similar behavior is observed for other temperature values. However, no such maximum is detected the 0–20 kOe range for the nanowire system (see inset Fig. 6(b)), indicating the absence of field-induced metamagnetic transition.

### Electrical Characterization

**Figure 7:**
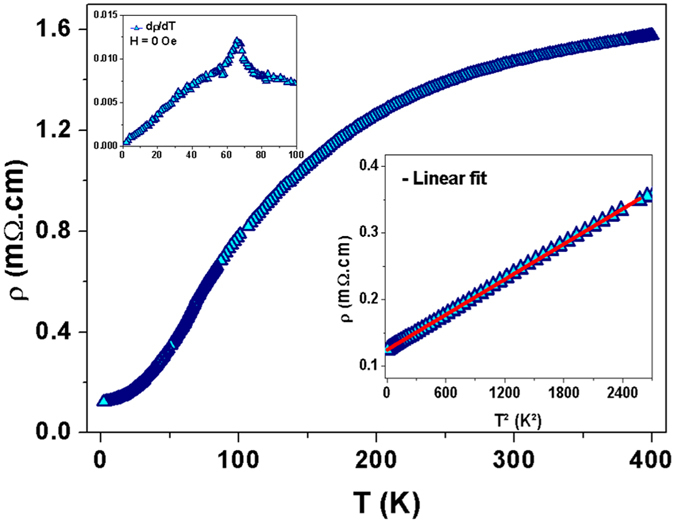
Temperature-dependent electrical resistivity for the Fe_3_Ga_4_ bulk compound (without magnetic field). Upper left inset: Maximum of the resistivity derivative versus temperature. Lower right inset: resistivity *vs T *^2^.

Bulk electrical resistivity measurements were performed using a standard four-probe technique in the 2–400 K temperature range and under magnetic fields of 0 and 7 kOe. Without applied magnetic field, temperature-dependent electrical resistivity curve exhibits a slope change at approximately 65 K (Fig. 7, see upper left inset). This temperature roughly corresponds to the bulk *T*_1_ value in temperature-dependent magnetization curve done under 500 Oe applied magnetic field. The linear dependence of resistivity as a function of squared temperature (Fig. 7, lower right inset) indicates that spin fluctuations play an important role at low temperature^30^,^31^. These results reveal that the resistivity of metamagnetic compounds is dominated by spin fluctuations, an important aspect that justifies the use of Moriya’s theory to explain the Fe_3_Ga_4_ compound magnetic behavior. With a magnetic field of 7 kOe, the temperature where the resistivity slope is changing, which happens to be 70 K, increases, in agreement with the magnetization results (not shown here).

### Magnetic Phase Diagram

**Figure 8:**
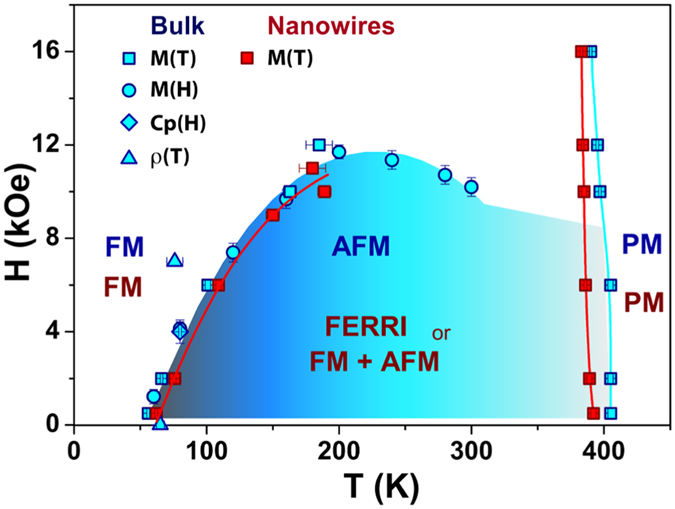
Magnetic phase diagram for Fe_3_Ga_4_ bulk and nanowires form showing the effect on the transition temperatures due to the lower dimensionality.

Experimental magnetic, specific heat and electrical results yield to the built magnetic phase diagrams of the Fe_3_Ga_4_ bulk and for the system at low dimensionality (Fig. 8). Based on our observations and Moriya and Usami’s theory, we have strong evidences that the effects of reducing dimensionality can be explained as resulting from modifications of the system free energy. Those modifications originate from electronic structure changes, and end up affecting the compound magnetic phase diagram. Thus, Fe_3_Ga_4_ bulk compound would exhibit at *T*_1_ either a ferromagnetic to antiferromagnetic transition (in agreement with the literature for bulk), or a ferromagnetic to ferrimagnetic or coexistence of ferromagnetic and antiferromagnetic transition, if under nanowire form. The specific heat data are also consistent with these conclusions, since a FERRI or FM behavior is most likely to be expected in localized electron magnetism. Finally, at *T*_2_, the applied field decreases the temperature of maximum susceptibility similarly for both systems.

## Conclusions

We have successfully grown both Fe_3_Ga_4_ bulk and nanowires by the new technique of metallic-flux nanonucleation. All the results suggest that, based on the Moriya’s theory, there is a transition from ferromagnetic to antiferromagnetic state for Fe_3_Ga_4_ bulk, while that for Fe_3_Ga_4_ nanowire array, there is a transition from ferromagnetic to ferrimagnetic or a coexistence of ferromagnetic and antiferromagnetic state. These changes in the nature and aspects of the magnetic transitions are supported to arise from electronic structure modifications, which can be tuned by controlling the system dimensionality. These results open the possibility for studying fundamental aspects of magnetic behavior of any intermetallic compound that can be fabricated by the metallic flux method, as well as could allow the design of new nanostructures used in nanometric devices with desired physical features.

## Methods

Bulk single crystals as well as nanowires specimens of Fe_3_Ga_4_ compound were synthesized through the innovating method of metallic-flux nanonucleation (MFNN). The MFNN technique is based on the conventional flux-growth technique^32^ performed in a nanoporous alumina template that mediates the preferential nucleation of the single crystals in the desired geometry. First, alumina templates were prepared via a hard anodization process^33^. High purity (99.999%) aluminum foils were degreased, cleaned, dried, thermally treated at 400 °C for 3 hours and finally electropolished. The anodization procedure was performed in a 0.3 M oxalic acid bath at 1 °C. After a first anodization performed at 40 V for 5 min, the anodization voltage was gradually increased until reaching 120 V at a 0.8 V/s rate, where it was kept for 2 hours. The obtained templates are 100 *μ*m thick, with 250 nm diameter pores arranged in a hexagonal array with 270 nm interpore distance. Both Fe_3_Ga_4_ compounds in bulk and nanowire shape were simultaneously obtained in the same bath using the Ga self-flux technique. For this procedure, appropriated quantities of high purity (99.995%) Fe and (99.999%) Ga precursors were deposited in an alumina crucible, together with the nanoporous alumina template properly fixed at the crucible bottom and sealed in a quartz tube under vaccum. In order to form Fe_3_Ga_4_ crystals via the flux-growth technique, the sealed tube was heated up to 1100 °C for 12 h, cooled down to 700 °C at 3 °C/h and finally cooled down to 400 °C at 8 °C/h. The filled pores embedded in the nanoporous alumina template were subsequently carefully separated from the bulk crystals.

Since the MFNN method yields a simultaneous synthesis of crystalline bulk and nanowires, any intermetallic compound that can be prepared by the flux-growth method can also be, in principle, synthesized in nanowire form. This represents an enormous advantage for the MFNN method.

## Additional Information

**How to cite this article**: Moura, K. O. *et al*. Dimensionality tuning of the electronic structure in Fe_3_Ga_4_ magnetic materials. *Sci. Rep.* **6**, 28364; doi: 10.1038/srep28364 (2016).
